# Serum Phospholipase A2-IIA, hs-CRP, and Lipids in Women With Subclinical Hypothyroidism

**DOI:** 10.5812/ijem.16967

**Published:** 2014-06-10

**Authors:** Mohammad Hossein Shojaei Nik, Masoud Darabi, Amir Ziaee, Fatemeh Hajmanoochehri

**Affiliations:** 1 Department of Biochemistry and Clinical Laboratories, International Branch (Aras), Tabriz University of Medical Sciences, Tabriz, IR Iran; 2 Liver and Gastrointestinal Disease Research Center, Faculty of Medicine, Tabriz University of Medical Science, Tabriz, IR Iran; 3 Metabolic Diseases Research Center, Qazvin University of Medical Sciences, Qazvin, IR Iran

**Keywords:** Hypothyroidism, Group II Phospholipases A2, Lipids, C-Reactive Protein

## Abstract

**Background:**

Subclinical hypothyroidism (SCH) is a metabolic disorder characterized by elevated TSH level but normal T4 level. Some previous studies suggest that SCH is associated with inflammation.

**Objectives:**

The present study aimed to compare lipid serum levels in SCH patients and normal participants, also explore possible association between SCH and the two inflammatory markers hs-CRP and PLA2-IIA.

**Patients and Methods:**

This study was performed on 77 women aged 20-45 (39 with SCH and 38 in the control group). TSH and T4 levels were measured by electrochemiluminescenceassay. Lipid profiles were analyzed using enzymatic-colorimetric methods. Hs-CRP and PLA2-IIA were determined using the ELISA method. IBM SPSS 19.0 was used for statistical analysis.

**Results:**

Serum levels of TG, cholesterol, and LDL were higher in the SCH group than the control group. However, there was no significant difference between the two groups for HDL level. Likewise, no difference was observed for the serum level of hs-CRP. PLA2-IIA mean value was higher in the SCH group.

**Conclusions:**

SCH is associated with increased level of PLA2-IIA, which is independent of BMI. The stronger association of SCH with PLA2-IIA than with hs-CRP indicates that PLA2-IIA is an inducer of inflammation while hs-CRP is not.

## 1. Background

Subclinical hypothyroidism (SCH) is a metabolic disorder characterized by elevated serum concentrations of thyroid-stimulating hormone (TSH) and normal serum concentrations of thyroxin (T4) (1, 2). Its prevalence in general population is 1-10%, but it nearer 15% in women, particularly the elderly (3, 4).

Hypothyroidism is one of the main causes of abnormal lipid metabolism (5, 6). Patients with overt hypothyroidism are at risk of hypertension, cardiovascular disease, and atherosclerosis (7). However, there is no general agreement about the association between these conditions and SCH, also no agreement on the necessity of hormone therapy in all SCH patients (8-12). Although the association between SCH and dyslipidemia is still controversial, changes in lipid profile in patients with SCH have been observed in several studies (13-16). Dyslipidemia leads to atherosclerosis via an inflammatory process, with oxidized low-density lipoprotein (LDL) playing an important role. Kvetny et al. (12) found a lower high-density lipoprotein (HDL) and higher triglyceride (TG) serum levels in patients with SCH in comparison with the control group, but they did not observe any significant difference in LDL and total cholesterol levels.

Some studies have investigated hs-CRP (high sensitive C-reactive protein) and phospholipase A2 (PLA2; EC 3.1.1.4) as markers of inflammation in patients with SCH. CRP is an acute-phase response protein, which is synthesized by liver cells. Trace levels of CRP are present in normal conditions, but CRP increases rapidly in response to inflammatory conditions (17). A weak association was reported by Kvetny et al. (12) between hs-CRP and SCH. In contrast, Hueston et al. (18) found no association between SCH and hs-CRP.

PLA2 is an acute-phase response protein present in different tissues and cellular secretions, including vascular smooth muscle cells, platelets, neutrophils, and hepatocytes. PLA2, as a lipolytic enzyme, has an important role in inflammation through hydrolysis of membrane phospholipids in the sn-2 position, thereby producing non-esterified fatty acids such as arachidonic acid and lysophospholipids. PLA2 is divided into four main types: secreted (sPLA2), cytosolic (cPLA2), calcium-independent (iPLA2), and lipoprotein-associated (lp-PLA2). Ten members of the sPLA2 family have been identified in mammals: GIB, IIA, IIC, IID, IIE, IIF, III, V , X and XII (19).

PLA2-IIA was isolated from synovial fluid of patients with rheumatoid arthritis (20). This type of PLA2 can modify LDL via hydrolysis and leads to the formation of smaller and denser LDL particles associated with increased risk of cardiovascular disease (21, 22). Serum concentrations of PLA2-IIA are low in normal conditions, but the levels significantly increase during acute or chronic inflammatory conditions (23, 24). PLA-A2 is an inflammatory marker like CRP, and there is a strong association between the two markers (25).

## 2. Objectives

The present study aimed to (a) compare lipid serum levels in SCH patients and normal participants and (b) explore possible association between SCH and the two inflammatory markers hs-CRP and PLA2-IIA.

## 3. Patients and Methods

### 3.1. Participants

Of all outpatients referred to a laboratory for thyroidal tests, only 77 were selected. Inclusion criteria were female gender, age between 20-45 years old, and fasting state. Outpatients who had a history or an evidence of serious problems or known diseases, particularly heart complaint, thyroid, diabetes mellitus, hypertension, and mental retardation and those who had been recently hospitalized or were taking medication and especially anti-inflammatory drugs were excluded from the study. In addition, pregnant, breastfeeding, or menopausal women and those who were taking contraceptive drugs were excluded from the study.

Selected participants were assigned to either the SCH group (39 patients) or the control group (38 members) according to the results of thyroidal tests. Both groups should have a T4 serum level ranging from 60 to 160 nmol/L. However, TSH serum level should be in the range of 5-10 µIU/mL for the experimental group and 0.3-5 µIU/mL for the control group.

The local ethical committee approved the study protocol. Informed consent was obtained from outpatients before participation. Body weights (Kg) and heights (cm) were measured, and BMI was measuredas weight per height squared (kg/m^2^). All blood samples were taken from antecubital vein after overnight fasting. Serum was separated from each blood sample after standard centrifugation. Serum samples were stored at -70°C.

### 3.2. Measurement

The electrochemiluminescence method (ECL) was used on Elecsys 2010 machine for measuring TSH and T4. The kits were purchased from Cobas Roche Diagnostics (Germany). The kit for TSH test had an intra-assay coefficient variation (CV) of less than 8.6%, an inter-assay CV of less than 8.7%, and sensitivity of 0.005 µIU/mL. T4 was measured using a kit of intra-assay CV of < 0.46%, inter-assay CV of < 0.54%, and sensitivity of 5 nmol/L.

Lipid profile was measured using the enzymatic-colorimetric method on Automated Biochemistry Analyzer Hitachi 917 (Japan) and by means of Bionic Diagnostic Kits (Iran). Kit characterization was as follows:

1. TG: inter-assay CV of < 7.7%, intra-assay CV of < 1.57%, and sensitivity of 5 mg/dL.
2. Cholesterol: inter-assay CV of < 6.9%, intra-assay CV of < 1.2%, and sensitivity of 5 mg/dL.
3. LDL: inter-assay CV of < 1.7%, intra-assay CV of < 0.6%, and sensitivity of 3 mg/dL.
4. HDL: inter-assay CV of < 1.5%, intra-assay CV of < 0.8%, and sensitivity of 2.5 mg/dL.

Enzyme-linked immunosorbent assay (ELISA) method was used for measuring hs-CRP and PLA2-IIA. The kits for hs-CRP and PLA2-IIA were obtained from Monobind Inc. (USA) and Bioassay and Technology Laboratory (China), respectively. The hs-CRP kit was as follows: intra-assay CV of < 7.8%, inter-assay CV of < 9%, and sensitivity of 0.014 µg/mL. The kit for PLA2-IIA had the following characteristics: inter-assay CV of < 12%, intra-assay CV of < 10%, and sensitivity of 2.36 pg/mL.

### 3.3. Statistical Analysis

SPSS 19.0 (IBM, USA, 2010) was used for statistical analysis. First, one-sample Kolmogorov-Smirnov Test was performed to determine normal distribution of data (data with Pvalue ≥ 0.5 considered normally distributed). Results were expressed as mean ± standard deviation. For variables that were not normally distributed, Wilcoxon-Mann-Whitney Test was performed, with the results being shown as median (min-max). Independent-samples t-test was used to compare the parameters between the two experimental and control groups. Binary logistic regression was used for adjusting the data. Statistical significance was set at P values ≥ 0.05 in all cases.

## 4. Results

Table 1 the results obtained by comparing the two groups of study. As can be seen, the mean ages in the two groups were not statistically different (P ≥ 0.05), but the mean BMI value was higher in the experimental group than the control group (P = 0.01). Furthermore, the mean T4 serum level was significantly lower in the SCH group than the other group (P = 0.01). Finally, Table 1 that all lipids except HDL were significantly higher in the experimental group compared tothe control group.

**Table 1.** Clinical Characteristics and Laboratory Findings in Patients With SCH and Control Subjects ^a^

Variables	Control, n = 38	SCH, n = 39	P value
**Age, y**	30.08 ± 5.86	33.00 ± 7.11	≥ 0.05
**BMI, kg/m** ^**2**^	24.10 ± 3.99	26.71 ± 4.69	0.01
**PLA2-IIA, pg/mL (min-max)**	50.15 (37.57-151.40)	55.21 (37.57-187.70)	0.01
**hs-CRP, µg/mL(min-max)**	0.084 (0.072-27/25)	1.04 (0.129-31.51)	≥ 0.05
**Cholesterol, mg/dL**	169.16 ± 28.51	186.62 ± 36.64	0.02
**TG, mg/dL (min-max)**	77 (27-178)	95 (44-217)	0.001
**HDL, mg/dL**	49.11 ± 9.09	48.33 ± 8.53	≥ 0.05
**LDL, mg/dL**	95.18 ± 25.15	110.95 ± 33.48	0.02
**TSH, µIU/mL**	2.87 ± 0.81	6.83 ± 0.94	0.001
**T4, nmol/L**	103.43 ± 14.13	94.23 ± 17.48	0.01

^a^ Abbreviations: BMI, body mass index; HDL, high density lipoprotein; LDL, low density lipoprotein; PLA2, group II phospholipases A2; SCH, subclinical hypothyroidism; TG, triglycerides; TSH, thyroid-stimulating hormone; T4, thyroxine.

**Figure 1.**
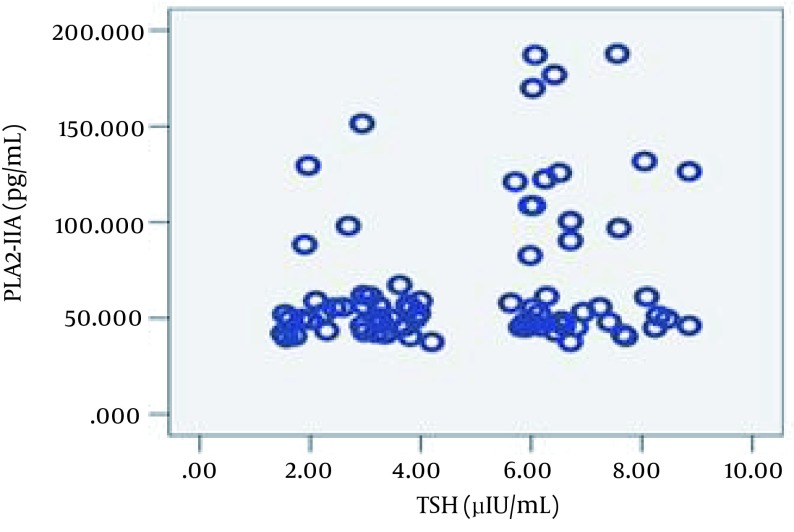
Correlation Between TSH and PLA2-IIA (ρ = 0.037, r = 0.239)

**Figure 2.**
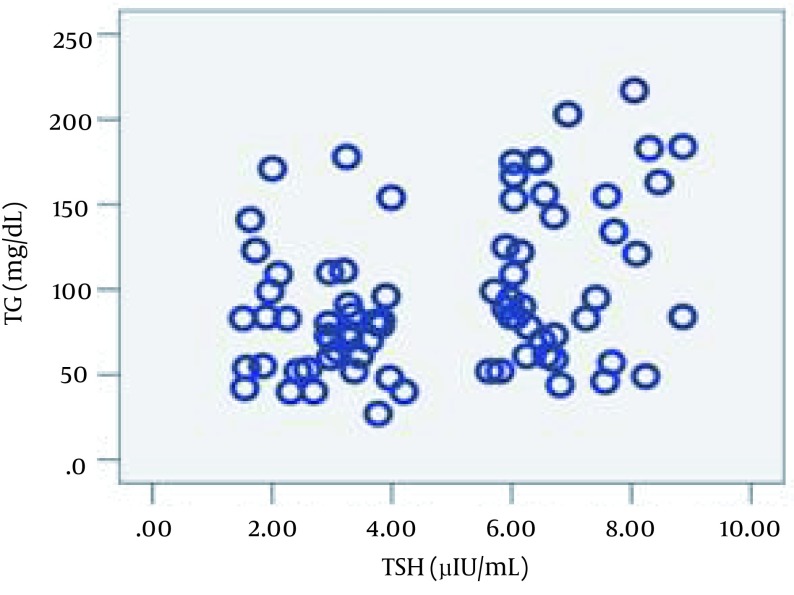
Correlation Between TSH and TG (ρ = 0.002, r = 0.344)

Regarding the inflammatory markers, the SCH group had higher mean PLA2-IIA and hs-CRP levels, although statistical significance was only observed in the former case. Subsequently, the Pearson product-moment correlation coefficient was calculated to measure linear dependence between the variables. The correlation observed between TSH and PLA2IIA (ρ = 0.037, r = 0.239) and between TSH and TG (ρ = 0.002, r = 0.344) was positive. Figures 1 and 2 a schematic representation of these correlations.

Now given the observation that the mean BMI value of the SCH patients was significantly more than that in the control group, we aimed to ensure the difference between the two groups f or the inflammatory markers not affected by BMI difference. For this purpose, binary logistic regression was performed, with the results given in Table 2. This data adjustment did not show any statistically significant effect for either BMI (P = 0.137), LDL (P = 0.649), TG (P = 0.066), or and cholesterol (P = 0.586). However, the association between SCH and PLA2-IIA was still significant (confidence interval: 1.005-1.040, P = 0.014), indicating that the association between the two was independent.

**Table 2.** Binary Logistic Regression of Clinical and Laboratory Findings in the Studied Groups ^a,b^

Variables	P value	OR	95% Confidence Intervals
**BMI, kg/m** ^**2**^	0.137	1.106	0.968-1.264
**LDL, mg/dL**	0.649	0.985	0.924-1.051
**TG, mg/dL**	0.066	1.013	0.999-1.027
**Cholesterol, mg/dL**	0.586	1.016	0.959-1.076
**PLA2-IIA, ng/dL**	0.014	1.022	1.005-1.040

^a^ Binary logistic regression was used for data adjustment.

^b^ Abbreviations: OR, Odd’s Ratio-; BMI, body mass index; LDL, low density lipoprotein; PLA2, group II phospholipases; TG, triglycerides.

## 5. Discussion

Findings of the present study support the association between SCH and dyslipidemia. Significantly higher serum levels of cholesterol, LDL, and TG in SCH group were in agreement with some previous studies (13-16) and yet contradicted some other studies (26, 27). For instance, a study in South Korea (28) showed that serum levels of total cholesterol and LDL were significantly higher in patients with SCH to that of normal individuals. Moreover, whereas the present research observed no significant difference between the experimental and control groups for HDL, some studies (13) reported a decrease in HDL serum levels in patients with SCH. Significantly, higher levels of PLA2-IIA and higher mean value of hs-CRP in the SCH group in the current research supported the hypothesis that SCH is associated with an inflammatory condition. The higher level of hs-CRP obtained here was also reported by Tuzcu et al. (15) and Christ-Crain et al. (29), which showed an increase in hs-CRP serum level in SCH patients, and also Kvetny et al. (12) in males with SCH below 50 years. However, Jung et al. (28) did not find any significant difference in hs-CRP level between SCH patients and the control group. The stronger association between SCH and PLA2-IIA than between SCH and hs-CRP indicates that PLA2-IIA is an inducer of inflammation whereas hs-CRP is not. The positive correlation between TSH and PLA2-IIA and between TSH and TG on the one hand, and higher mean values of cholesterol, LDL, and hs-CRP in the SCH patients on the other hand support the hypothesis that SCH may increase the risk of atherosclerosis. Hak et al. (30) showed that elderly women with SCH are more at risk of atherosclerosis and myocardial infarction, and Rodondi et al. (31) reported an increased risk of cardiovascular disease (CVD) in SCH patients. Moreover, Erem (32) showed an increase in the activity of factor X as well as a more atherogenic lipid profile in SCH patients, attributing to increased risk of atherosclerosis complications in these patients. In contrast, there are some studies which disagree. For example, Hueston et al. (18) showed no difference between SCH and normal people regarding serum levels of hs-CRP and homocysteine. Further, a meta-analysis (33) concluded that there was no adequate evidence for accepting this hypothesis. High level of PLA2-IIA is not only a mediator for localized inflammation, but also predicts adverse outcomes in patients with CAD (34). Milionis et al. (16) confirmed the associationbetween SCH and atherosclerosis by evaluating lp-PLA2. However, we did not find any other study regarding the association between PLA2-IIA and SCH. It remains to be elucidated whether the presence of an inflammatory process in SCH patients is directly or indirectly (via lipids) caused by mild thyroid dysfunction. If we considerthe “direct” interpretation, thyroid hormone deficiency at cellular level may induce a lack of energy and may lead to a chronic intracellular inflammatory process.
